# Regulation of the Tumor Suppressor FOXO3 by the Thromboxane-A_2_ Receptors in Urothelial Cancer

**DOI:** 10.1371/journal.pone.0107530

**Published:** 2014-09-09

**Authors:** Philip M. Sobolesky, Perry V. Halushka, Elizabeth Garrett-Mayer, Michael T. Smith, Omar Moussa

**Affiliations:** 1 Department of Pathology and Laboratory Medicine, Medical University of South Carolina, Charleston, South Carolina, United States of America; 2 Hollings Cancer Center, Medical University of South Carolina, Charleston, South Carolina, United States of America; 3 Departments of Pharmacology and Medicine, Medical University of South Carolina, Charleston, South Carolina, United States of America; 4 Department of Public Health Sciences, Medical University of South Carolina, Charleston, South Carolina, United States of America; University of Kentucky College of Medicine, United States of America

## Abstract

The transcription factor FOXO3 is a well-established tumor suppressor whose activity, stability, and localization are regulated by phosphorylation and acetylation. Previous data by our laboratory demonstrated amplified thromboxane-A_2_ signaling was associated with poor prognoses in bladder cancer patients and overexpression of the thromboxane-A_2_ isoform-β receptor (TPβ), but not TPα, induced malignant transformation of immortalized bladder cells *in vivo*. Here, we describe a mechanism of TP mediated modulation of FOXO3 activity and localization by phosphorylation and deacetylation in a bladder cancer cell model. *In vitro* gain and loss of function studies performed in non-transformed cell lines, UROsta and SV-HUC, revealed knockdown of FOXO3 expression by shRNA increased cell migration and invasion, while exogenously overexpressing TPβ raised basal phosphorylated (p)FOXO3-S294 levels. Conversely, overexpression of ERK-resistant, mutant FOXO3 reduced increases in UMUC3 cell migration and invasion, including that mediated by TP agonist (U46619). Additionally, stimulation of UMUC3 cells with U46619 increased pFOXO3-S294 expression, which could be attenuated by treatment with a TP antagonist (PTXA_2_) or ERK inhibitor (U0126). Initially U46619 caused nuclear accumulation of pFOXO3-S294; however, prolonged stimulation increased FOXO3 cytoplasmic localization. U46619 stimulation decreased overall FOXO3 transcriptional activity, but was associated with increased expression of its pro-survival target, manganese superoxide dismutase. The data also shows that TP stimulation increased the expression of the histone deacetylase, SIRT1, and corresponded with decreased acetylated-FOXO3. Collectively, the data suggest a role for TP signaling in the regulation of FOXO3 activity, mediated in part through phosphorylation and deacetylation.

## Introduction

Bladder cancer is the fifth most prevalent cancer in the United States with superficial transitional cell carcinoma (TCC) being the most commonly diagnosed form [1]. TCC has a high recurrence rate and requires costly lifelong follow-ups, making bladder cancer one of the most expensive cancers to treat over a patient’s lifetime [2]. Understanding the mechanisms of oncogenic transformation of urothelial cells is essential to designing effective and novel therapies for the treatment of bladder cancer. We previously identified an inverse association between thromboxane synthase (TXS) expression and survival of bladder cancer patients, suggesting a role for thromboxane prostaglandin (TP) signaling in urothelial tumor progression [3]. TXS is an important enzyme that catalyzes the conversion of prostaglandin H_2_ to thromboxane A_2_ (TXA_2_). The TXA_2_ ligand, in turn, activates the TP receptor, promoting cell migration, proliferation, and invasion, which are key hallmarks of cellular transformation and the progression of disease [3], [4], [5], [6], [7]. Other studies have indicated a role for TXA_2_ signalling in the tumorigenesis and malignant phenotypes of prostate [8] and breast cancers [9], [10].

The human TP receptor gene encodes two isoforms, TPα and TPβ [11]. Both isoforms are seven trans-membrane G protein coupled receptors that differ only at the C-terminal domain [12]. The C-terminal variation allows each isoform to interact with both identical and unique signaling mediators [13]. Expression of the TP receptor isoforms is tissue and cell-type dependent. We previously reported TPβ overexpression in bladder cancer patients was associated with a significant decrease in survival [14]. In addition, the overexpression of TPβ, but not TPα, was sufficient to increase migration, invasion, and proliferation, as well as induce the malignant transformation of an immortalized non-transformed urothelial cell line [14]. The mechanism of transformation induced by TPβ overexpression remains unknown.

The transcription factor forkhead box-O3 (FOXO3) regulates key cell survival processes including oxidative stress resistance, cell cycle arrest, and apoptosis through the regulation of its target genes e.g. manganese superoxide dismutase (MnSOD), p27^Kip1^, Fas ligand (FasL), and Bim [15], [16]. FOXO3 transcriptional activity can be regulated through post-translational modifications (PTM) such as acetylation and phosphorylation. Dysregulation of FOXO3 activity and localization has been associated with cancer initiation and progression, as well as chemotherapeutic resistance [17], [18], [19]. Acetylation of FOXO3 by the histone acetyl-transferase p300 has been linked to decreased FOXO3 activity, increased cytoplasmic localization, and degradation [20]. Deacetylation by NAD-dependent deacetylase Sirtuin-1 (SIRT1) has been shown to differentially regulate FOXO3 targets by promoting the transcription of p27^Kip1^ and MnSOD, while decreasing transcription of Bim and FasL [21]. Extracellular signal-regulated kinase 1 and 2 (ERK1/2) has been shown to phosphorylate FOXO3 at -S294, -S344, and -S425, and negatively affect its function and stability through murine double minute 2 (MDM2)-mediated FOXO3 degradation [22]. While stimulation of TPβ is known to induce phosphorylation and activation of ERK [23], [24], [25], to our knowledge no study has examined the effects of TP signaling on FOXO3 modulation.

Here, we identified FOXO3 as a downstream target of the TP receptor pathway and examined the negative effects of TP signaling on FOXO3 localization and function in the bladder-cancer derived cell line UMUC3. Interestingly, overall FOXO3 transcriptional activity was reduced following TP agonist stimulation resulting in reduced expression of an apoptotic target, yet enhanced expression of a stress resistance target. In accordance with previous studies [21], the differential expression of FOXO3 targets following TP agonist stimulation was associated with increased SIRT1 expression and deacetylated FOXO3. Taken together, this study elucidates a mechanism by which TP induced modulation of FOXO3 activity through post-translational modifications contributes to the malignant phenotype of urothelial cells.

## Materials and Methods

### Cell culture

The UROsta cell line, kindly provided by Dr. Donald Sens (University of North Dakota, Grand Forks, ND) was derived from normal human urothelial cells and immortalized with the SV40 T-antigen [26]. UROsta cells were maintained in a 70∶30 mixture of DMEM medium (1 g/L glucose: 4.5 g/L glucose; Mediatech, VA, USA), 10% fetal bovine serum (FBS). The Simian virus 40-immortalized human uroepithelial (SV-HUC), nontransformed urothelial cell line was kindly provided by Dr. Santhanam Swaminathan (University of Wisconsin, Comprehensive Cancer center, Madison, WI) [27], [28], [29] and cells were cultured in F12K Khaigns modified media supplemented with 10% FBS, insulin, hydrocortisone, and transferrin. The bladder cancer cell line UMUC3 were obtained from American Type Culture Collection and cells cultured in RPMI 1640 medium (Thermo Fisher Scientific, Rockford, IL, USA) containing 10% FBS. Every media contained 1% penicillin/streptomycin. Cell lines were grown at 37°C in 5% CO_2_.

### Antibodies, reagents, and plasmids

The following antibodies were used according to the manufacturers’ recommendations: pFOXO3-S294, p-c-Jun Ser63, c-Jun, pAKT Ser473, pMAPK Thr202/Tyr204, Akt, ERK, p300, Sirt1, Bim, p27^Kip1^ (Cell Signaling Technology, Danvers, MA, USA), FOXO3, GFP (Santa Cruz), Lamin B1 (GeneTex, Irvine, CA, USA) MnSOD (EnzoLife Sciences, Ann Arbor, MI, USA), α-Tubulin (AbD Serotec, Raleigh, NC, USA), GAPDH (Sigma-Aldrich, St. Louis, MO, USA). TP specific agonist, U46619 was purchased from Cayman Chemicals (Ann Arbor, MI, USA). ERK1/2 inhibitor U0126 was purchased from Sigma-Aldrich. FHRE-luc plasmid was a gift from Michael Greenberg (Addgene plasmid #1789, Cambridge, MA, USA). The GFP-FOXO3^3A^ (Serines-294, -344, and -425 substituted to alanines) and control pEGFP-C_2_ plasmid were gifts from Mien-Chie Hung at The University of Texas M.D. Anderson Cancer Center. The GFP-TPβ plasmid was a gift from Jean-Luc Parent from the University of Sherbrooke, Canada.

### Western blot analysis

Cells were washed with PBS and harvested in RIPA buffer (Thermo Fisher Scientific) supplemented with Complete Protease Inhibitors Cocktail Tablets and PhosSTOP Phosphatase Inhibitors Cocktail Tablets (Roche Applied Science). Proteins were denatured by boiling in Laemmli buffer (BioRad, Hercules, CA, USA) and resolved by 12% SDS-PAGE. Following protein transfer, the nitrocellulose membrane was blocked with 5% milk in 1× TBST and probed with primary antibody in 5% BSA in 1× TBST overnight at 4°C. Following primary antibody incubation, membranes were washed in 1× TBST and incubated with HRP-linked secondary antibody (Cell Signaling Technology) in 1× TBST. Membranes were washed 5× for 5 min with 1× TBST and detection was visualized using SuperSignal West Pico Chemiluminescent Substrate (Thermo Fisher Scientific). Western blots were quantified using Image Studio Lite Ver. 3.1 following software instructions and graphed on GraphPad Prism 5.

### Transfections and generation of stable clones

To generate stable FOXO3 knockdown clones, SV-HUC and UROsta cells were transfected with either pRFP-C-RS control plasmid, pRFP-C-Scrambled or pRFP-c-shRNA to FOXO3 plasmid (HuSH-29; OriGene, Rockville, MD, USA) using FuGENE 6 (Roche Applied Science, Indianapolis, IN, USA). Single clones were selected in media containing 2.5 µg/ml puromycin (Invivogen, San Diego, CA, USA) and knockdown was analyzed by immunoblotting using a FOXO3 specific antibody (H-144; sc-11351; Santa Cruz Biotechnology, Santa Cruz, CA, USA). Transfection of GFP-TPβ into SVHUC cells was performed with TurboFect transfection reagent (Thermo Fisher Scientific) using a ratio of 1.5 µg DNA to 4 µl TurboFect. UROsta cells (1×10^6^) were electroporated with 3.5 µg GFP-TPβ using the Amaxa Cell Line Nucleofector kit V solution box (cat#: VCA-1003, Lonza, Allendale, NJ, USA) and the Nucleofector 2b device (Lonza) with the pre-programmed protocol T-030. Four hours following electroporation media was aspirated, replaced with complete media, and incubated at 37°C in 5% CO_2_. Transfection efficiency was checked 24 hours post-electroporation. To generate stable GFP-FOXO3^3A^ expressing UMUC3 clones, cells in a 6-well plate were transfected using 4 µl X-tremeGENE HP DNA Transfection Reagent (Roche Applied Science) and 1.5 µg plasmid DNA. Single clones were selected in media containing 500 µg/ml of Geneticin Selective Antibiotic (Life Technologies, Grand Island, NY, USA).

### Cell migration assay

The BD falcon Cell Culture Insert System containing polyethylene terephthalate (PET) membranes with 8 µm pores (BD Biosciences, Rockville, MD, USA) were used for this assay. The inserts were pre-coated overnight with 5 µg/cm^2^ fibronectin at 4°C and equilibrated in water for 2 hours at 37°C prior to usage. Trypsinized cells were collected, counted, and 5×10^4^ cells were resuspended in 250 µl serum-free medium. The upper chamber of the insert was filled with the cell suspension, whereas 750 µl of medium containing 10% FBS were added to the bottom of each well. UROsta cells were allowed to migrate for 16 hours in a humidified environment at 37°C with 5% CO_2_. After migration, cells were removed from the upper surface of the membrane by wiping it with a moist cotton swab. The lower surface of the membranes were stained using the Hema-3 Staining kit (Fisher Scientific, Pittsburg, PA, USA). Once the staining was finished the membranes were rinsed in distilled water for excess stain removal and air dried overnight. Percent migration or invasion was calculated by counting and averaging the number of cells invaded in 10 random fields of view at a 40× magnification, divided by the area of the microscope viewing field and then multiplied by the area of the transwell insert. Then divide the previously calculated number by the number of cells seeded and lastly multiply by 100 to get a percent. This experiment was performed independently three times in duplicates.

### Cell invasion assay

The BD BioCoat Matrigel Invasion Chamber Cell Culture Insert System containing 8 µm PET membrane (BD Biosciences) with a thin layer of MATRIGEL Basement Membrane Matrix was used for this assay. Trypsinized cells were collected, counted, and resuspended at 5×10^4^ cells/ml in serum-free media. The top chamber received 500 µl of the cell suspension, while 750 µl of RPMI with 10% FBS was added to the well. The cells were allowed to invade for 16 hours in a humidified environment at 37°C with 5% CO_2_ before cell removal from the upper surface with a moist cotton swab. Membranes were stained using the Hema-3 Staining kit (Thermo Fisher Scientific) and percent invasion was calculated as described in cell migration assay section. This experiment was performed independently three times in duplicates.

### Dual luciferase assay

UMUC3 cells were seeded in 6-well plates at 0.3×10^6^ cells/well, then transiently transfected within 24 hours using X-tremeGENE HP DNA transfection reagent (Roche Applied Science) with 4 µg of 3× forkhead response element (FHRE) reporter construct (Addgene plasmid #1789) and 0.1 µg pBind Renilla luciferase control vector (Promega, Madison, WI, USA). Transfected cells were incubated for 24 hours in a humidified environment at 37°C with 5% CO_2_. Cells were then serum starved overnight and treated with either vehicle control (methyl acetate, Sigma-Aldrich) or 1 µM U46619 (Cayman Chemical) for 30 or 120 minutes. Following treatment cells were washed twice with PBS and harvested in Reporter Lysis Buffer (Promega). Luminescence was measured using a Veritas Microplate Luminometer (Turner BioSystems, Sunnyvale, CA, USA) in 20 µl of each sample for 10 seconds following each injection of 50 µl Luciferase Assay Reagent II then 50 µl Stop and Glow. The average Firefly luciferase expression was adjusted to total protein and control vector Renilla luciferase expression, and then normalized to vehicle control. Results represent the average percent activity of three independent experiments conducted in duplicates, Student *t*-test (*) P<0.05.

### Nuclear/cytoplasmic cell fractionation

UMUC3 cells were serum starved overnight, then pretreated with 1 µM Indomethacin (Cayman Chemicals) for 10 minutes to reduce endogenous TXA_2_ signaling. Cells were treated with vehicle or 1 µM U46619 for 5, 30, or 120 minutes. Cells were trypsinized, collected, and washed twice with PBS. Fractionation was performed using the Pierce NE-PER Nuclear and Cytoplasmic Extraction Kit (Thermo Fisher Scientific) following the manufacturer’s protocol. Extracts were analyzed by Western blot.

### Immunoprecipitation

Immunoprecipitation of FOXO3 from UMUC3 cells was performed using the NHS-linked IP/Co-IP kit (Thermo Fisher Scientific) following manufacturers protocol. Briefly, the UMUC3 cells were grown to 80% confluence in 10 cm plates, and then placed in serum free media for 24 hours in a humidified environment at 37°C with 5% CO_2_. Cells were then treated with either vehicle control (methyl acetate, Sigma-Aldrich) or 1 µM U46619 (Cayman Chemical) for 30, 60, or 120 minutes. Following treatment cells were washed once with PBS (−Ca, −Mg) and harvested in ice cold IP lysis/wash buffer supplemented with protease inhibitor EDTA-free cocktail and PhosSTOP inhibitor cocktail (Roche). Collected lysate then centrifuged to remove cell debris and determined protein concentration using BCA protein assay kit (Thermo Fisher Scientific). The NHS-activated magnetic beads were vortexed and 25 µl of slurry was transferred to 1.5 mL tubes per IP reaction. Tubes were placed on the magnet and storage solution was discarded, and then washed with ice-cold 1 mM HCl and gently vortexed for 5 seconds. Beads were collected on magnetic stand and discarded the supernatant.

The antibody solution was prepared by diluting two µg FOXO3 (Santa Cruz Biotechnology) and negative control rabbit immunoglobulin fraction (solid-phase absorbed) (Dako, Carpinteria, CA, USA) to a final concentration of 2 µg antibody in 100 µL of 0.067M borate buffer. Added 100 µl of prepared antibody solution to the activated beads, gently vortexed, and incubated on a rotating platform for 60 minutes at room temperature. Reactions were vortexed every 10 minutes during the incubation to ensure the beads remained in suspension. Beads were collected and supernatant was discarded. Washed beads twice with elution buffer, vortexing tubes each wash to remove non-covalently bound antibody. Quenching buffer was added to the beads and incubated on a rotating platform for 60 minutes at room temperature. Beads were collected on magnetic stand and discarded the supernatant. Prepared and added 0.5 mL of modified borate buffer to each IP and mixed gently by vortex, then beads were collected on magnetic stand and discarded the supernatant. Reactions were washed twice with IP lysis/wash buffer, then incubated each IP reaction with 100 µg lysate in final volume of 500 µL IP lysis/wash buffer supplemented with protease and phosphatase cocktail inhibitors overnight (between 14–18 hours at 4°C on a rotator. The beads were vortexed every 15 minutes the first hour to ensure the beads stayed in suspension. After antigen incubation the beads were collected, washed twice with IP lysis/wash buffer and then transferred to a new 1.5 ml tube. Washed beads with ultrapure water (pH 7.0), then placed on magnetic stand and discarded the supernatant. Proteins were eluted from the beads with low pH elution buffer (pH 2.0) twice and neutralized with Tris-HCl pH 8.5. Eluted proteins were then analyzed by Western blot.

### Statistical analysis

Continuous outcomes were compared across conditions using a two-sided two-sample Student’s *t*-test. Experiments containing more than two variables were compared using a one-way ANOVA followed with a Tukey post-hoc test. A P-value <0.05 was considered statistically significant.

## Results

### Knockdown of FOXO3 Increases Urothelial Migration and Invasion

**Figure 1:**
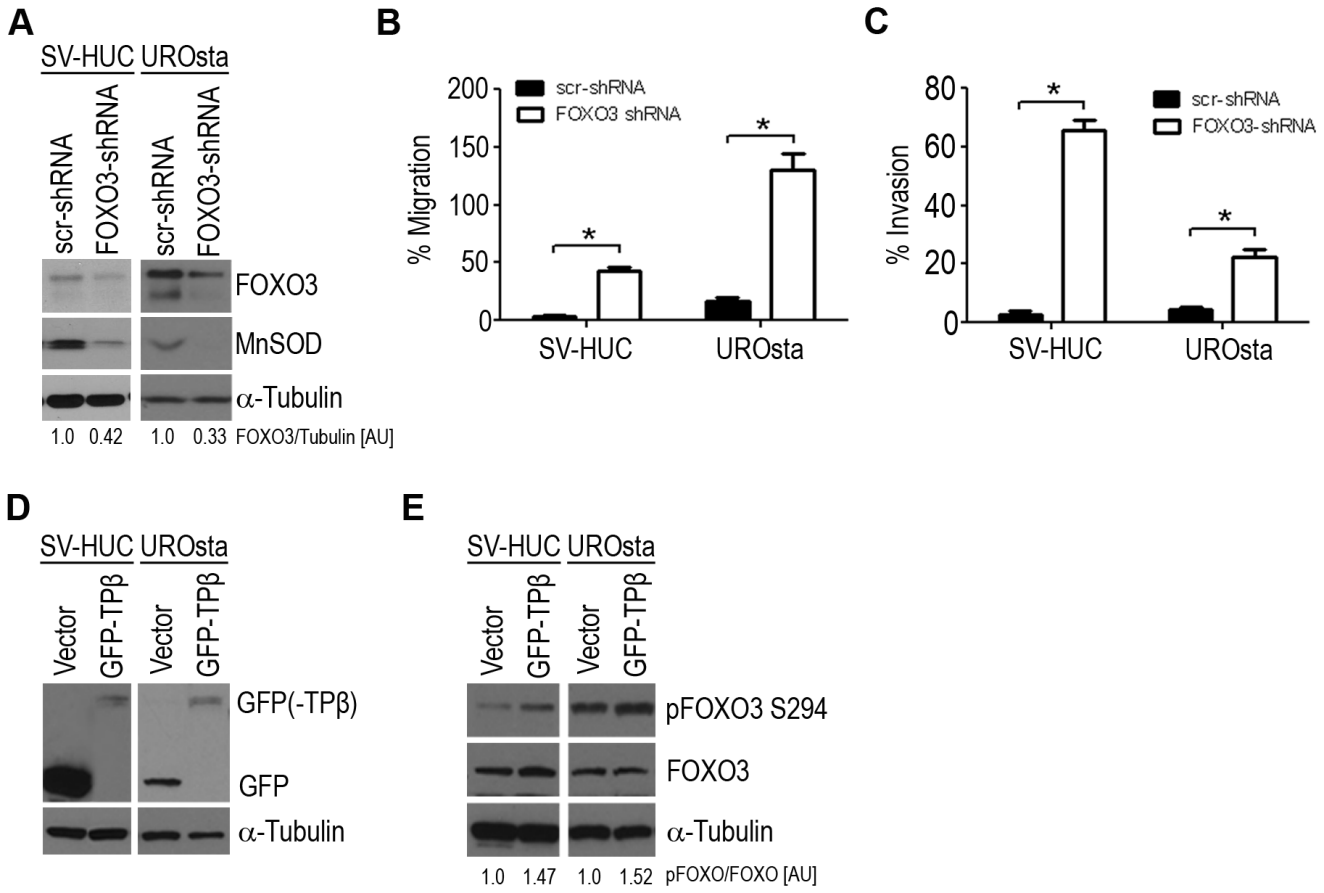
Loss of FOXO3 results in increased cell migration and invasion. (**A**) Western blot of whole cell lysates from SV-HUC and UROsta cells stably transfected with FOXO3-shRNA or control scrambled (scr−) shRNA immunoblotted for total FOXO3 and its known downstream target MnSOD. α-tubulin was applied as loading control. Blots shown are representative of three individual experiments and numbers represent the averaged ratios of FOXO3 to α-tubulin. Ratios quantified using Image Studio Lite Ver.3.1. SV-HUC and UROsta FOXO3 knockdown and control clones were seeded into inserts coated with fibronectin or matrigel and allowed to migrate (**B**) or invade (**C**) for 16 hours. Knockdown of FOXO3 increased SV-HUC and UROsta cell migration (39% and 114%) and invasion (63% and 18%, respectively) compared to scr-shRNA. Data represent three independent assays done in duplicates and expressed as the mean ± SEM. Significance was determined with a two-sided two-sample *t*-test, (* = P<0.05, n = 3) compared to scr- control. (**D**) Overexpression of GFP tagged TPβ in UROsta and SVHUC cells increases basal pFOXO3-S294 expression (**E**) compared to vector control (n = 3). Numbers represent the averaged ratios of pFOXO3-S294 to FOXO3. Ratios quantified using Image Studio Lite Ver.3.1. [AU] = arbitrary units.

We previously reported a role for the TPβ receptor in urothelial migration, invasion, and malignant transformation [3], [14]. Reduced FOXO3 expression in patients has also been linked with urothelial cancer invasiveness [30]. To determine whether FOXO3 inactivation would be sufficient to induce a malignant phenotype independent of TPβ, low endogenous TPβ expressing UROsta and SVHUC cell lines (previously determined [14]) were stably transfected with FOXO3 specific shRNA (Figure 1A). FOXO3-shRNA transfected cells reduced FOXO3 protein expression over one half compared to scrambled (scr)-shRNA observed by Western blot and validated by examining the downstream target MnSOD (Figure 1A). Compared to scr-shRNA cells knockdown of FOXO3 expression resulted in a 39% and 114% increase in migration, as well as a 63% and 18% increase in invasion of SV-HUC and UROsta cells, respectively, (P<0.05, Figure 1B–C). Since the absence of FOXO3 was sufficient to induce a malignant phenotype in immortalized non-transformed urothelial cells, we examined the effect of TPβ overexpression on FOXO3 inactivation. SV-HUC and UROsta cells transfected with TPβ displayed a 0.5-fold increase in the basal expression of the inactive pFOXO3-S294 protein (Figure 1D–E).

### Effects of TP Agonist Stimulation on FOXO3 Phosphorylation, Localization, and Transcriptional Activity

**Figure 2:**
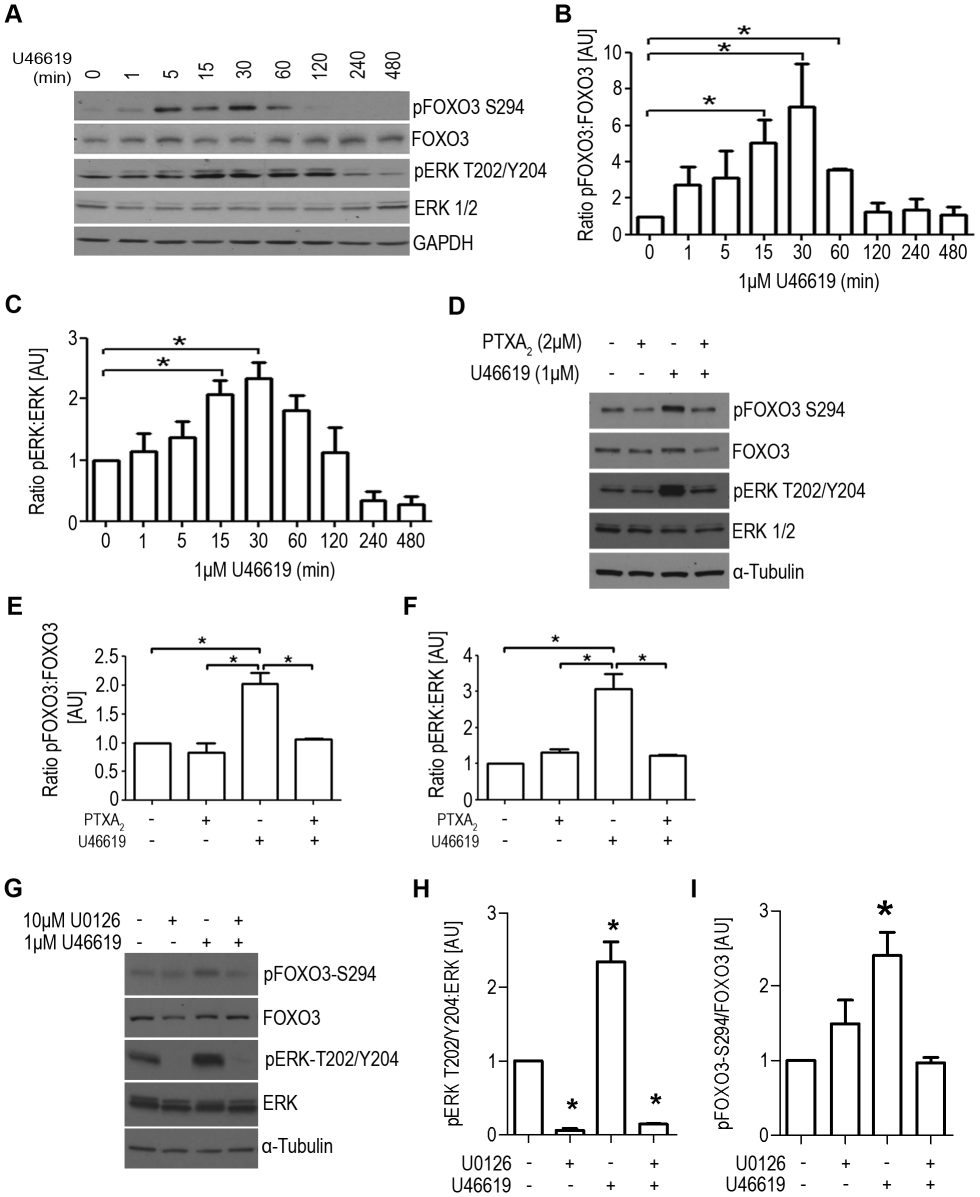
TP agonist activates ERK and increases FOXO3 phosphorylation at –S294. (**A**) UMUC3 cells were treated with 1 µM U46619 for the indicated times. Cell lysates were analyzed by immunoblotting for both total and phosphorylated ERK and FOXO3. GAPDH served as loading control. Blots were quantified and ratios for (**B**) pFOXO3-S294 to FOXO3 and (**C**) pERKT202/Y204 to ERK1/2 were graphed using GraphPad Prism 5. UMUC3 cells pretreated for 15 minutes with (**D**) 2 µM TP antagonist Pinane (PTXA_2_) or (**G**) 10 µM ERK inhibitor (U0126) attenuated the activation of ERK and phosphorylation of FOXO3 following treatment with 1 µM U46619 for 30 minutes. Cell lysates were immunoblotted for both total and phosphorylated ERK and FOXO3. α-Tubulin served as loading control. Graphs represent quantified ratios for (**E** and **I**) pFOXO3-S294 to FOXO3 and (**F** and **G**) pERKT202/Y204 to ERK1/2 were graphed. Significance was determined for all quantified blots using a repeated measure One-way ANOVA with Tukey post-hoc test. P<0.05.

To demonstrate TXA_2_ signaling is involved in FOXO3 regulation, the phosphorylation status of FOXO3 and its known regulator ERK were examined in UMUC3 cells after TP agonist U46619 stimulation (Figure 2A). The TP agonist mediated phosphorylation of ERK at T202/Y204 was significantly increased (∼1-fold) 15 and 30 minutes following stimulation (Figure 2A and C). Correspondingly, a significant increase in pFOXO3-S294 was observed 15 (5-fold), 30 (7-fold), and 60 (3.5-fold) minutes following TP agonist stimulation (Figure 2A–B). Treatment of UMUC3 cells with the TP antagonist pinane (PTXA_2_) or ERK inhibitor (U0126) attenuated the U46619 mediated effects of activated ERK and pFOXO3-S294 (Figure 2D–I).

**Figure 3:**
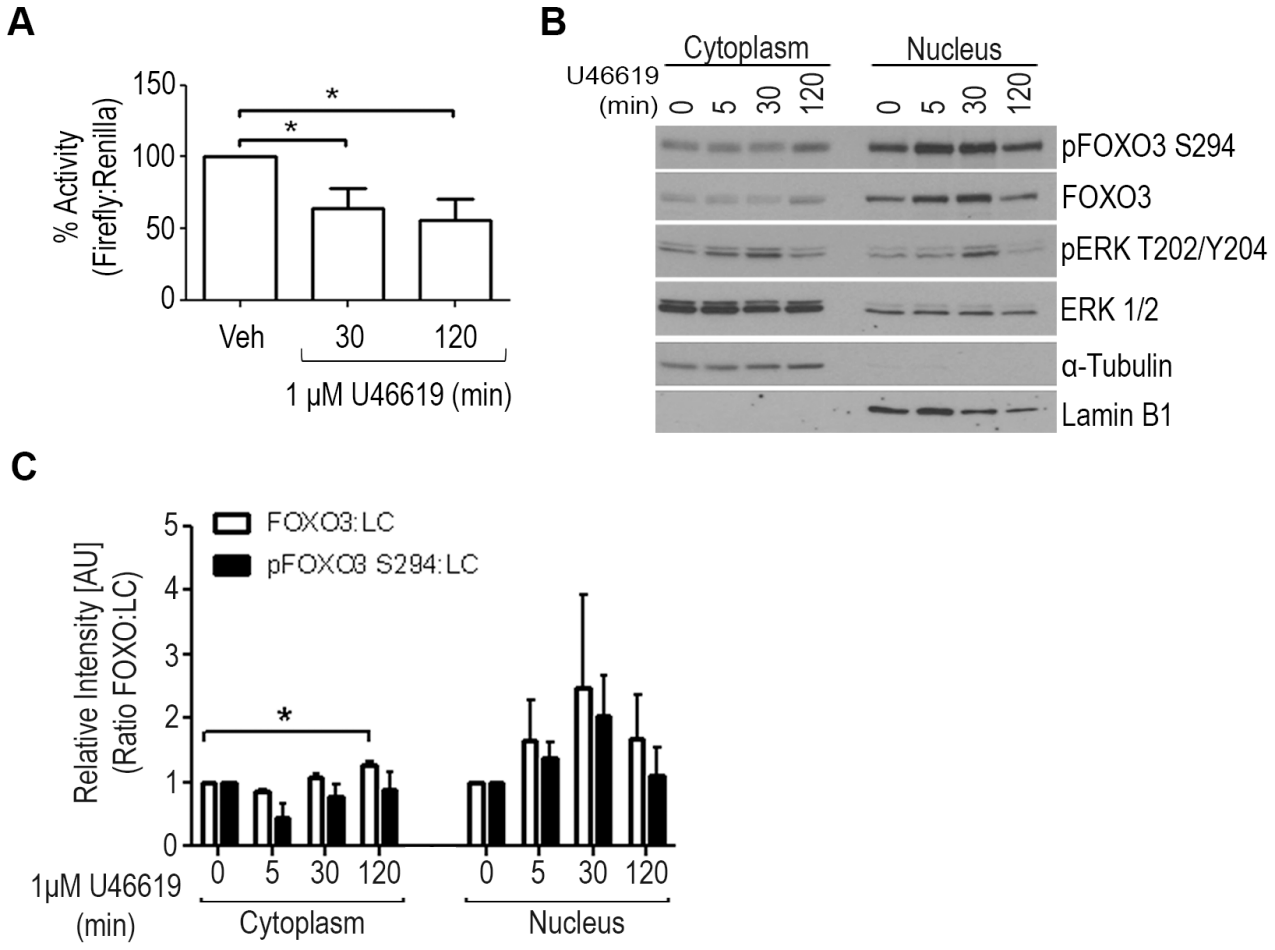
Decreased FOXO3 transcriptional activity and nuclear localization following TP agonist treatment. (**A**) UMUC3 cells transiently transfected with 3× forkhead response element (FHRE) reporter construct and Renilla luciferase control vector were treated with 1 µM U46619 for 30 or 120 minutes and resulted in decreased FOXO3 transcriptional activity. The average Firefly luciferase expression was adjusted to total protein and control vector Renilla luciferase expression before normalization to vehicle control. Results represent the average percent luciferase activity of three independent experiments conducted in duplicates. *P<0.05, One-way ANOVA with Tukey post-hoc test. (**B**) UMUC3 cells were serum starved overnight and pretreated for 15 min. with 1 µM Indomethacin prior to treatment with either vehicle or 1 µM U46619 for 5, 30, or 120 minutes. The cells were subjected to nuclear and cytoplasmic fractionation and proteins were analyzed by Western blot. α-Tubulin and Lamin-B1 served as loading controls for cytoplasmic and nuclear fractions, respectively. (**C**) Densitometry of blots showing prolonged agonist stimulation increased cytoplasmic FOXO3 protein and increased nuclear pFOXO3 proteins compared to vehicle controls. Significance was determined for all quantified blots using a repeated measure One-way ANOVA with Tukey post-hoc test. P<0.05.

The effects of TP agonist stimulation on FOXO3 transcriptional activity were examined by a dual luciferase assay. UMUC3 cells were transiently transfected with the forkhead response element (FHRE) firefly luciferase reporter construct and Renilla control vector, prior to treatment with vehicle or TP agonist. FOXO3 transcriptional activity was significantly decreased by 36% and 44% at 30 and 120 minutes, respectively, following TP agonist stimulation compared to vehicle control (Figure 3A).

Based on the phosphorylation kinetics of FOXO3, we examined the cellular distribution of FOXO3 in UMUC3 cells treated with vehicle or TP agonist for 5, 30, or 120 minutes. Nuclear and cytoplasmic protein fractions were analyzed by Western blot (Figure 3B) and band densities were quantified using Image Studio Lite Ver 3.1 (Figure 3C). UMUC3 cells stimulated with TP agonist showed an initial increase in nuclear FOXO3 which was also associated with an increase in nuclear pFOXO3-Ser294 (Figure 3C). Prolonged stimulation resulted in a significant increase in total FOXO3 in the cytoplasm (Figure 3C).

### TP stimulation increases SIRT1 expression, deacetylation of FOXO3, and affects FOXO3 target expressions

**Figure 4:**
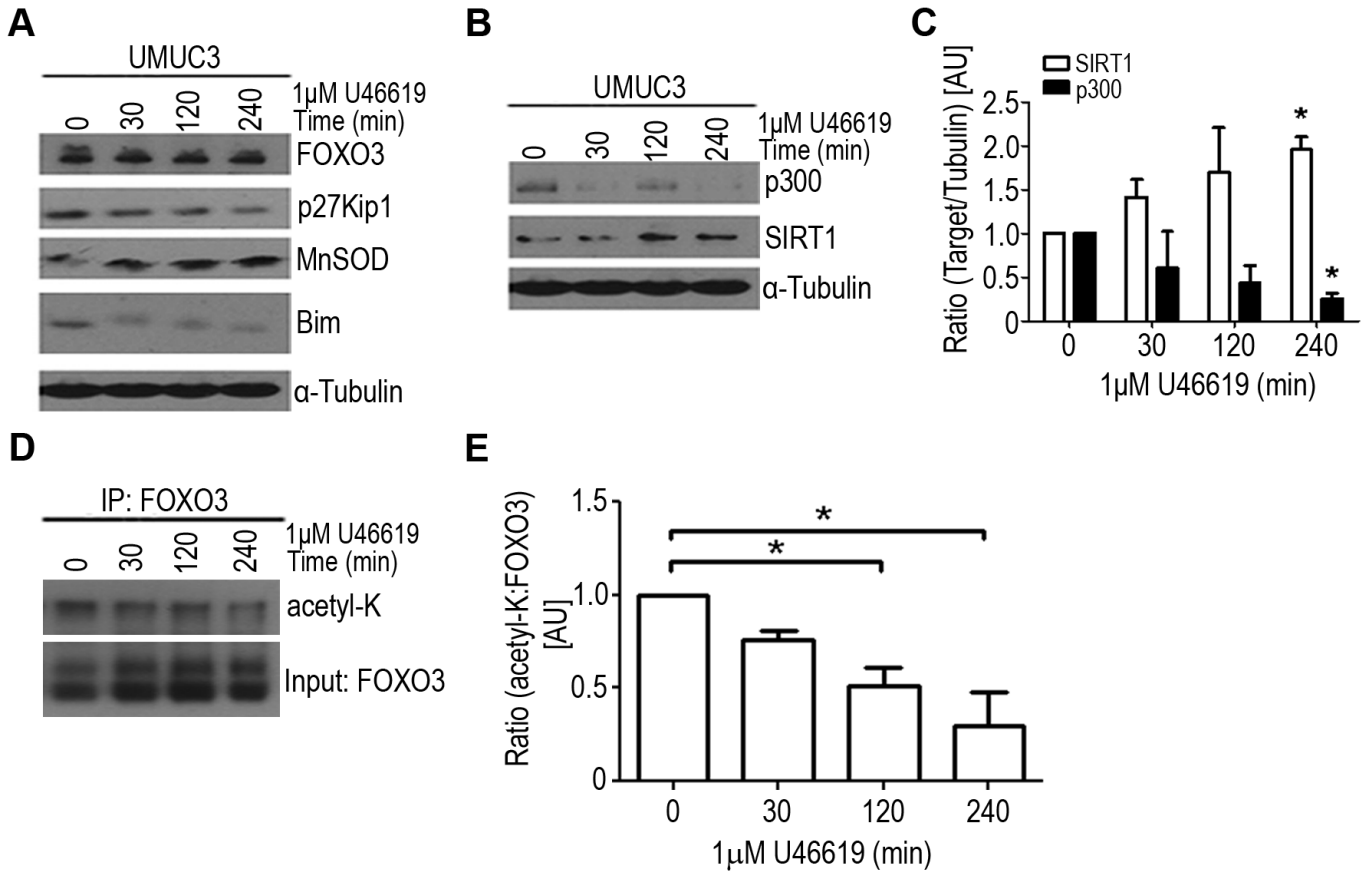
Effect of TP agonist on FOXO3 target proteins expressions and acetylation status. (**A**) UMUC3 cells treated with vehicle or 1 µM U46619 for 30, 120, and 240 minutes. Cell lysates were collected and analyzed by Western blot for total FOXO3 expression and downstream targets p27^Kip1^, MnSOD, and Bim. (**B**) Protein expression of p300 and SIRT1 in same cell lysates. α-tubulin served as loading control. (**C**) Quantified values from blots of three independent experiments are graphed. (*) indicates p<0.05, One-way ANOVA, compared to time 0 for each Target ratio. (**D**) Increased SIRT1 expression mediated by U46619 in UMUC3 cells corresponded with decreased acetylated FOXO3. Total FOXO3 was immunoprecipitated from serum starved UMUC3 cells treated with vehicle or 1 µM U46619 for 30, 120, or 240 minutes. Eluted proteins were analyzed by Western blot with antibodies against acetylated lysine residues and FOXO3. Blots are representative of three independent experiments. Blots were quantified and ratios for (**E**) acetylated-lysine (acetyl-K) to FOXO3 were graphed. Significance was determined using repeated measures One-way ANOVA with Tukey post-hoc test. P<0.05.

The effect of U46619 stimulation on the expression levels of well characterized FOXO3 targets p27^Kip1^, MnSOD, and Bim were examined by Western blot (Figure 4A). UMUC3 cells stimulated with U46619 for 30, 120, and 240 minutes observed decreased p27^Kip1^ and Bim proteins, and increased MnSOD protein expression (Figure 4A). Since the expression pattern of FOXO3 targets resembled the pattern observed following overexpression of the histone deacetyltransferase SIRT1 [21], we investigated the effect of U46619 stimulation on SIRT1 expression. UMUC3 cells stimulated with TP agonist displayed increased expression of SIRT1 and decreased expression of p300 compared to vehicle control (Figure 4B–C). To determine if the increased SIRT1 expression in UMUC3 cells stimulated with TP agonist affected FOXO3 acetylation status, FOXO3 was immunoprecipitated from UMUC3 cells treated with either vehicle or U46619, and immunoblotted for acetylated lysine residues. Stimulation of UMUC3 cells with U46619 for 120 and 240 minutes displayed a 48% and 70% reduction in acetylated FOXO3, respectively (Figure 4D–E).

### ERK resistant Mutated FOXO3^3A^ Reduced TP agonist Mediated Migration and Invasion

**Figure 5:**
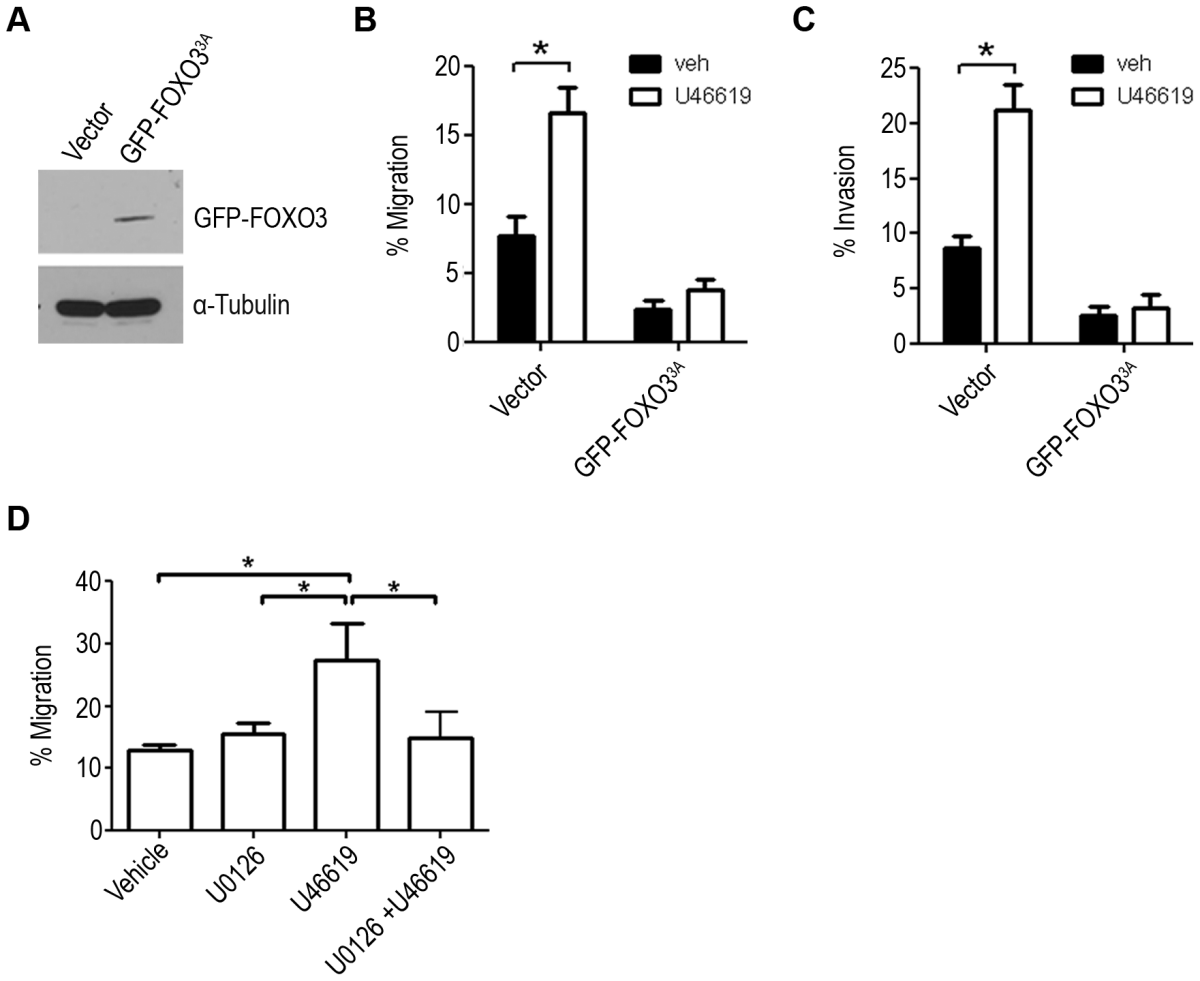
Expression of mutant FOXO3^3A^ attenuated TP agonist mediated migration of UMUC3 cells. (**A**) GFP-FOXO3^3A^ or EGFP-C_2_ (Vector) was stably transfected into UMUC3 cells and mutant FOXO3 expression was confirmed by immunoblotting for GFP. α-tubulin served as loading control. Cells were seeded into inserts coated with fibronectin for migration or matrigel for invasion in serum free media containing 1 µM U46619 and placed in wells containing complete media with 1 µM U46619. Cells were allowed to migrate (**B**) for 8 hours or invade (**C**) for 16 hours. Significance was determined with a two-sided two-sample *t*-test, (* = P<0.05, n = 3) compared to GFP-vector control. (**D**) UMUC3 cells were pretreated with 10 µM U0126 for 15 minutes before stimulation with 1 µM U46619 and allowed to migrate for 16 hours. Data represent three independent assays done in duplicates and expressed as the mean ± SEM. Significance was determined with a One-way ANOVA with Tukey post-hoc test. P<0.05.

UMUC3 cells were stably transfected with a GFP-vector or mutated GFP-FOXO3^3A^ construct in which all three ERK phosphorylation residues were substituted for alanine residues to mimic a non-phosphorylated or active state (Figure 5A). To determine if U46619 mediated phosphorylation of FOXO3 by ERK affects cell migration and invasion, the stably transfected cells were allowed to migrate and invade following stimulation with vehicle or U46619. The control GFP-vector cells resulted in a 2-fold increase in cell migration and invasion with U46619 stimulation compared to vehicle control, whereas expression of the GFP-FOXO3^3A^ protein reduced U46619 mediated migration and invasion (Figure 5B–C).

To further demonstrate the importance of ERK signalling in TXA_2_ mediated cell mobility, we pre-treated with the ERK inhibitor U0126 (10 µM) for 10 minutes before measuring the effect on U46619 mediated migration. UMUC3 cells pre-treated with U0126 did not significantly reduce basal levels of migration (Figure 5D). Pre-treatment with U0126 significantly reduced the U46619 induced cell migration of the UMUC3 cells (Figure 5D).

## Discussion

The current study supports the role of TXA_2_ signaling in promoting bladder tumorigenesis by modulating the activity of the transcription factor FOXO3 via ERK mediated phosphorylation at –S294 or deacetylation, presumably by SIRT1. We demonstrate the specificity of the effects on FOXO3 are mediated through the TXA_2_ pathway by using a pharmacological approach that includes a TP receptor(s) specific agonist and antagonist as well as an ERK inhibitor. We show shRNA mediated knock-down of FOXO3 in immortalized non-transformed bladder cells results in malignant phenotypes exhibited thru increases in cell migration and invasion. Thus, elucidating the effects of TXA_2_ signaling on FOXO3 expression, phosphorylation, and subcellular localization could reveal new chemotherapeutic targets for bladder cancer treatment.

TXA_2_ non-preferentially signals through the two alternatively spliced TP receptor isoforms, TPα (343 residues) and TPβ (407 residues), for the reason that the first 328 amino acid residues are identical [31]. Due to the short half-life of TXA_2_ (t½∼30 s), we used the TXA_2_ stable analogue U46619 at a previously established pharmacological concentration [14], [32], [33], [34]. While it is not possible to equate the concentration of U46619 to an *in vivo* concentration, the K_d_’s for TXA_2_ and U46619 are in a similar range and when TXA_2_ is generated it is often synthesized in large amounts so this concentration although pharmacologic could be considered to be in a physiologic range.

Stimulation of TPβ is known to signal through several G-proteins as well as β-arrestin2 to activate multiple pathways resulting in the activation of ERK [14], [35], [36], [37]. Our previous data identified the TPβ receptor isoform as overexpressed in the majority of bladder cancer cases as well as implicated its expression in bladder carcinogenesis and tumor progression [14]. Furthermore, the over-expression of TPβ, but not TPα, in immortalized bladder cells resulted in sustained ERK activation and induced malignant transformation in an in vivo mouse model [38], yet the mechanism for this effect remained unclear. Here, we showed overexpression of TPβ was sufficient to increase the phosphorylation of FOXO3 at Ser-294 implicating the ERK signaling pathway in the TXA_2_ mediated regulation of FOXO3. As a result, a goal of this research was to focus on the effects of TXA_2_ signaling on ERK mediated regulation of FOXO3 in a malignant cell line that expresses TPβ. To select the best cell line for examining the levels of modified FOXO3 protein, we immunoblotted a panel of bladder cancer cell lines for the expression of TPβ and FOXO3 and chose the cell line that expressed the highest levels of both proteins (data not shown). Consequently, the UMUC3 cell line was selected for examining modified FOXO3 protein levels. Moreover, the expression of a mutant FOXO3 protein that is unable to be phosphorylated by ERK, attenuated TP agonist mediated migration and invasion of malignant UMUC3 cells. We also demonstrated that inhibiting ERK pharmacologically is sufficient to attenuate the TP agonist induced migration of bladder cancer cells. Multiple studies have demonstrated the inactivation of FOXO3 by ERK is associated with cell survival and tumorigenesis of multiple cancers [22], [39], [40], however, this study is the first to experimentally link TXA_2_ signaling as the source of the effects on FOXO3.

The forkhead transcription factors are emerging as key players in cellular proliferation, migration, apoptosis, adaptation to cellular stress [39], and angiogenesis [41], [42], [43], [44], [45]. The subcellular localization of transcription factors can often determine their functionality, potentially making location as important as its overall expression in cancer. For instance, in prostate cancer increased cytoplasmic FOXO3 was associated with increased Gleason grade [19]. In ovarian cancer cells, the overexpression of phosphorylated FOXO3 at Thr-32 correlated with lymph node involvement [46]. In urothelial cancer, decreased FOXO3 expression was associated with increased invasiveness and decreased overall patient survival [30]. While those studies identified the importance of FOXO3 expression in patient survival, this study highlights the fact that the localization of FOXO3 and its phosphorylation status may be equally as important in predicting patient outcome.

Since loss or inactivation of FOXO3 activity is associated with tumor progression, drug resistance, and decreased patient survival in multiple cancers [19], [46], [47], [48], [49], we investigated the impact of FOXO3 protein loss on cellular migration and invasion of two independent immortalized non-transformed urothelial cell lines, UROsta and SV-HUC. The expression of FOXO3 was silenced with shRNA resulting in increased cell migration and invasion. To implicate the TPβ receptor in affecting FOXO3 function via phosphorylation, we exogenously overexpressed TPβ and observed increased pFOXO3-S294 expression. A key role of ERK phosphorylation in TXA_2_ mediated migration was previously demonstrated in human adipose tissue-derived mesenchymal stem cells pretreated with the ERK inhibitor U0126, which abrogated the U46619-induced cell migration, proliferation, and expression of α-smooth muscle actin [32], Similarly, we showed that the pharmacological inhibition of ERK with U0126 or the expression of a mutated FOXO3 that cannot be phosphorylated or inactivated by ERK in UMUC3 cells resulted in decreased TP agonist mediated migration and invasion. Moreover, pre-treatment with the TP antagonist PTXA_2_ attenuated U46619 induced activation of ERK. These data indicate that FOXO3 protein is negatively regulating TP mediated cell mobility of bladder cancer cells in part through phosphorylation by ERK. This suggests that antagonizing the TXA_2_ pathway could be an ideal therapeutic target in bladder cancer in part through its ability to prevent FOXO3 modulation.

Agonist stimulation of numerous G-protein coupled receptors (GPCR) has been shown to phosphorylate FOXO3, but result in differential FOXO3-induced cellular effects. For instance, cardiomyocytes treated with hypertrophic GPCR agonists such as angiotensin II, phenylephrine, isoproterenol, and IGF-1 have been shown to phosphorylate FOXO3 and promote its nuclear exclusion, resulting in cellular hypertrophy [50]. Whereas, we demonstrated in a bladder cancer cell model, the TP agonist U46619 induced FOXO3 phosphorylation in a time dependent manner (Figure 2A) and was concurrent with increased activated ERK1/2. The agonist mediated increase in pFOXO3-S294 and pERK1/2 is prevented with TP receptor antagonist PTXA_2_ which support the specificity of TP activation on FOXO3 phosphorylation. To further prove that the effect is specific to ERK1/2 activity, cells were incubated with the ERK inhibitor (U0126). U0126 treatment abolished U46619 mediated increase in both pFOXO3-S294 and pERK1/2 (Figure 2G–I). Although the fold changes in FOXO3 phosphorylation appear modest they were statistically significant and determined to be biologically relevant via its ability to affect U46619 mediated migration and invasion of urothelial cells. Furthermore, this suggests a more complex and potentially cell type dependent mechanism of FOXO3 tumor suppression that may require multiple posttranslational modifications of FOXO3 for the various GPCR mediated effects. Thus, future studies should be aimed at elucidating the signaling mediators upstream of the kinases that phosphorylate FOXO3 which affect its function and localization in response to GPCR stimulation.

Resistance to cisplatin, a common chemotherapeutic drug used to treat bladder cancer, has been associated with decreased FOXO3 expression in urothelial cell lines [18], [51]. Interestingly, cisplatin treatment increases renal synthesis of TXA_2_, which can lead to impaired renal function [52], [53], [54]. The use of TP antagonists have been shown to enhance the chemotherapeutic effects of cisplatin [14]. Future studies are needed to examine if the TP antagonist enhanced cisplatin effects are mediated through the regulation of FOXO3.

Dysregulation of oxidative stress resistance genes has been shown to promote carcinogenesis and tumor progression in a variety of cancers [55]. A study in high-grade and advanced stage bladder tumors consistently revealed an inverse relationship in the expression of FOXO3 targets, MnSOD and catalase, [56], which could result in increased cellular hydrogen peroxide levels. Furthermore, deacetylation of FOXO3 by SIRT1 has been reported to have a dual effect on the expression of FOXO3 targets such that SIRT1 expression increases expression of p27^Kip1^ and MnSOD, but inhibits the expression of Bim [21], [57]. In this study, a similar FOXO3 target expression pattern was observed following TP agonist stimulation in cells known to endogenously overexpress TPβ [14]. We found TP agonist stimulation increased SIRT1 expression and corresponded with decreased acetylated FOXO3 and increased MnSOD protein. Moreover, TPβ has been shown to be stabilized by hydrogen peroxide [58], the byproduct of MnSOD. This suggests a possible autocrine feedback loop that occurs with elevated TP signaling, such that TP activation results in the specific upregulation of FOXO3 transcriptional target MnSOD which could promote TPβ stability via elevated hydrogen peroxide levels.

Despite numerous studies that have linked decreased TXA_2_ synthesis via cyclooxygenase inhibitors with better patient outcomes [59], [60], [61], [62], [63], [64], there are currently no ongoing clinical trials in the United States that are investigating the effects of TP specific antagonists as adjuvant therapy for the treatment of cancer. In conclusion, we have demonstrated a novel mechanism of FOXO3 regulation through phosphorylation and acetylation mediated by the thromboxane A_2_ signaling pathway in bladder cancer. A better understanding of the TXA_2_ mediated mechanism of FOXO3 regulation in urothelial cells will promote the identification of new therapeutic strategies such as directly targeting the TP receptors with antagonists. Future studies will be focused on elucidating the differences between the signaling mediators for each TP receptor isoform and determining which mediators facilitate the activation of the kinases responsible for regulating FOXO3 activity.
